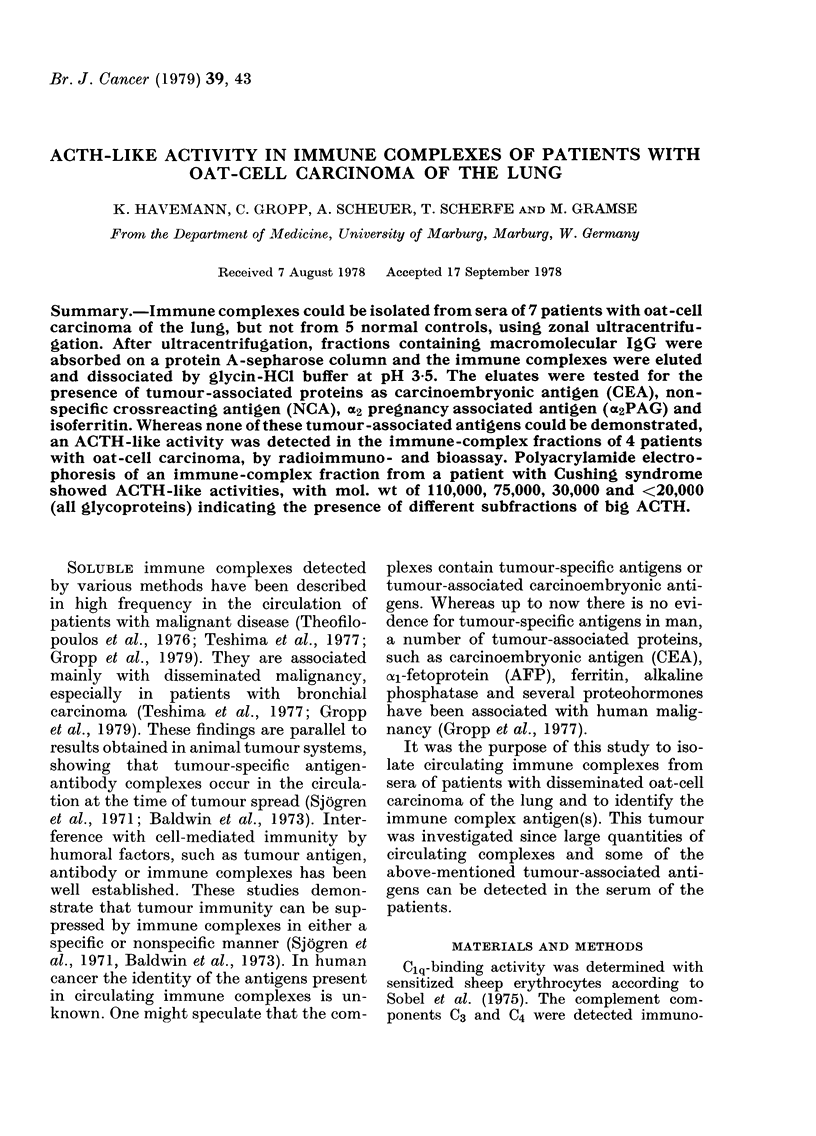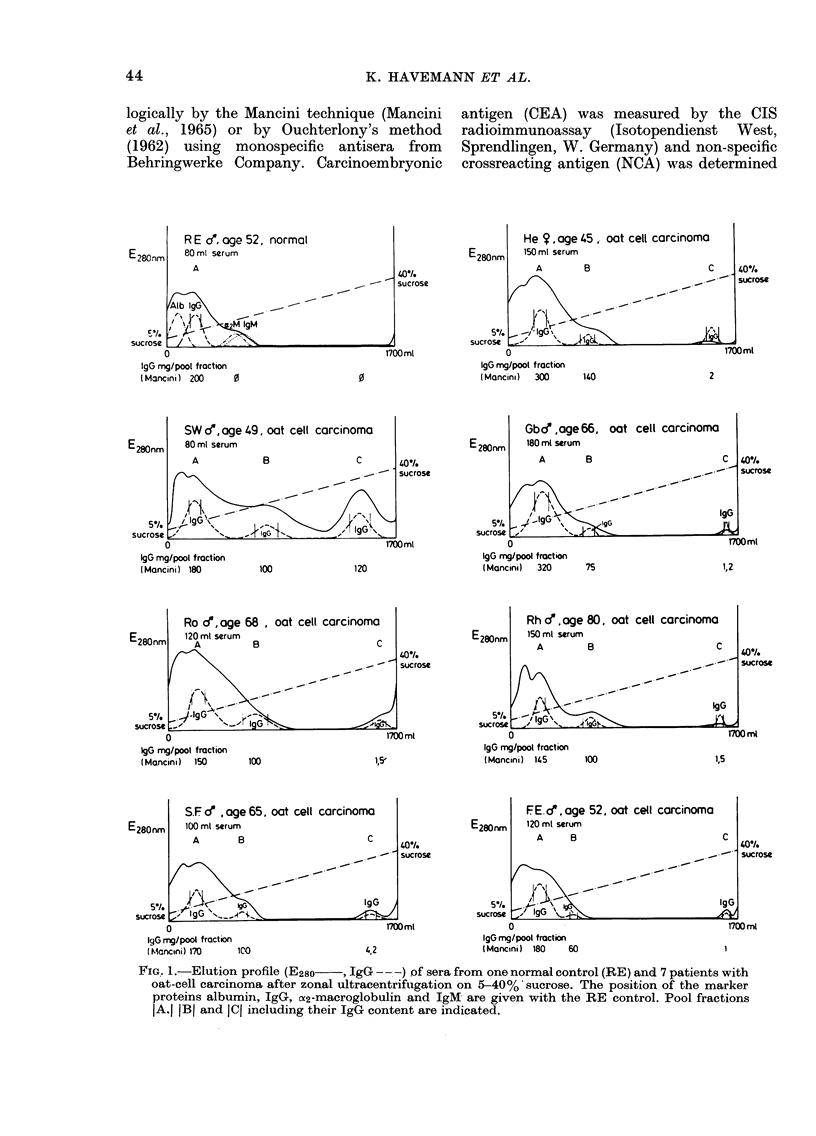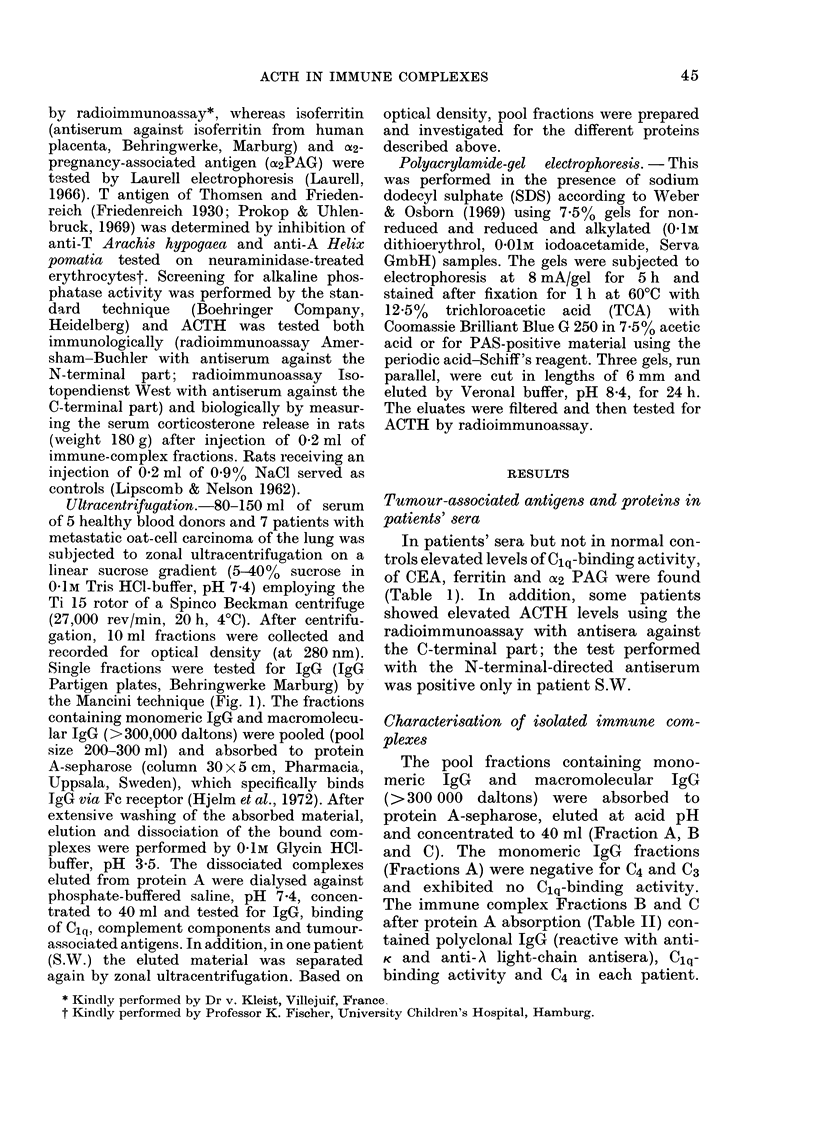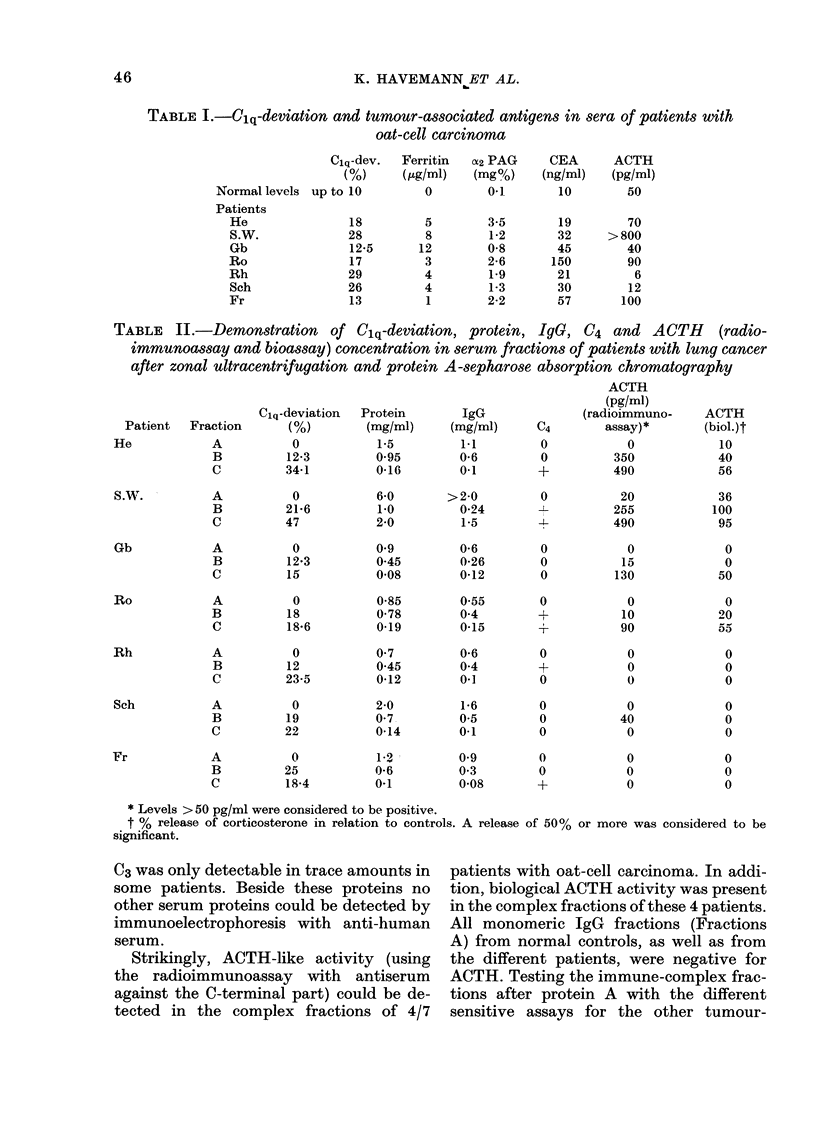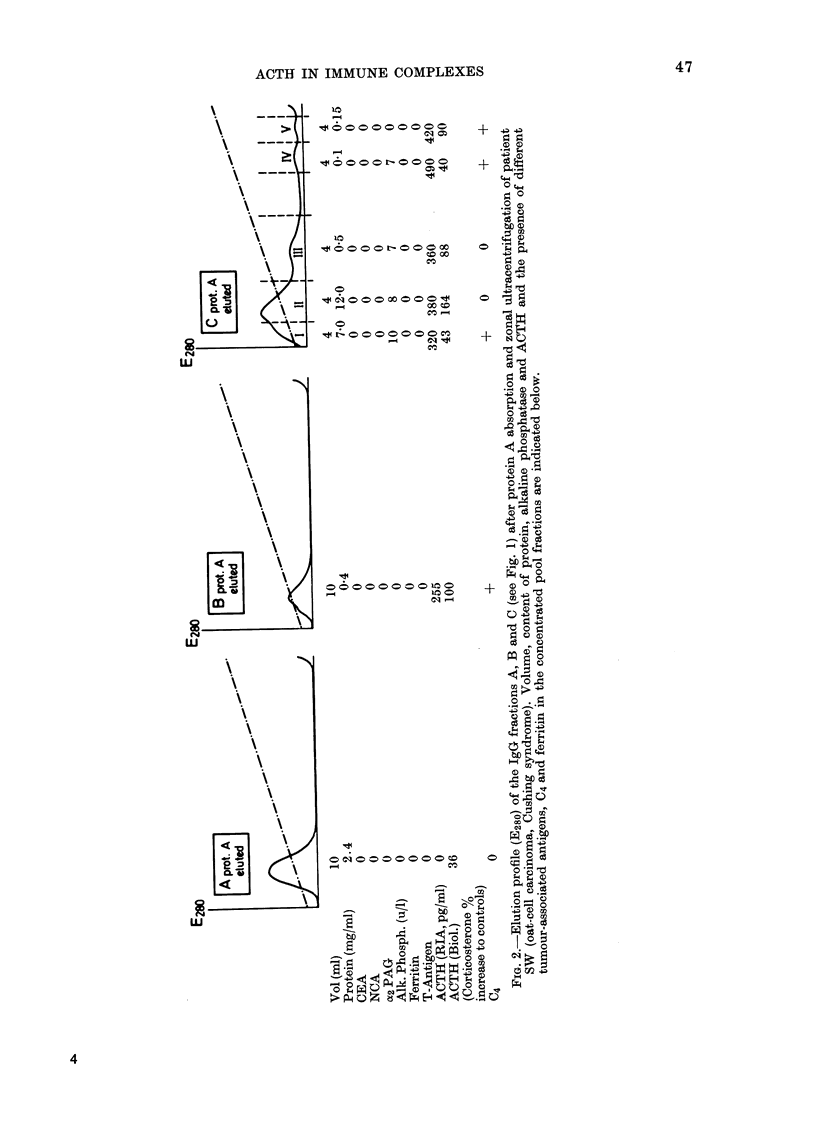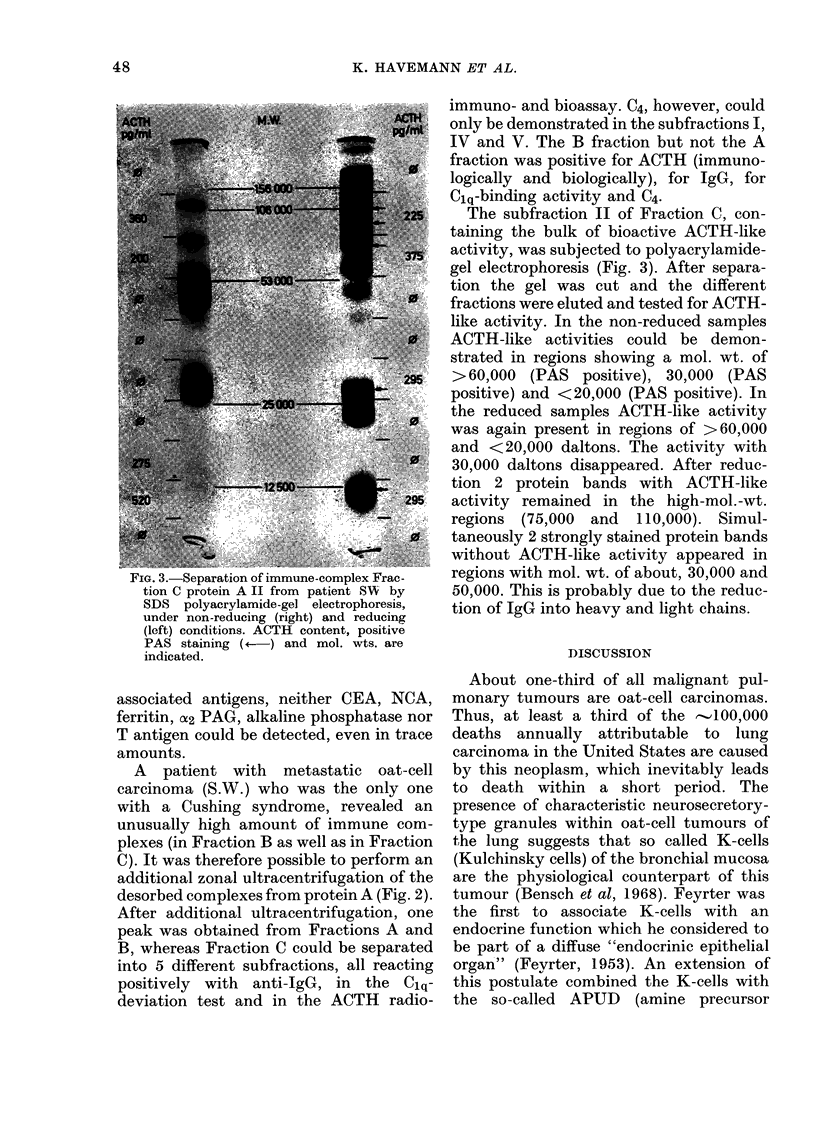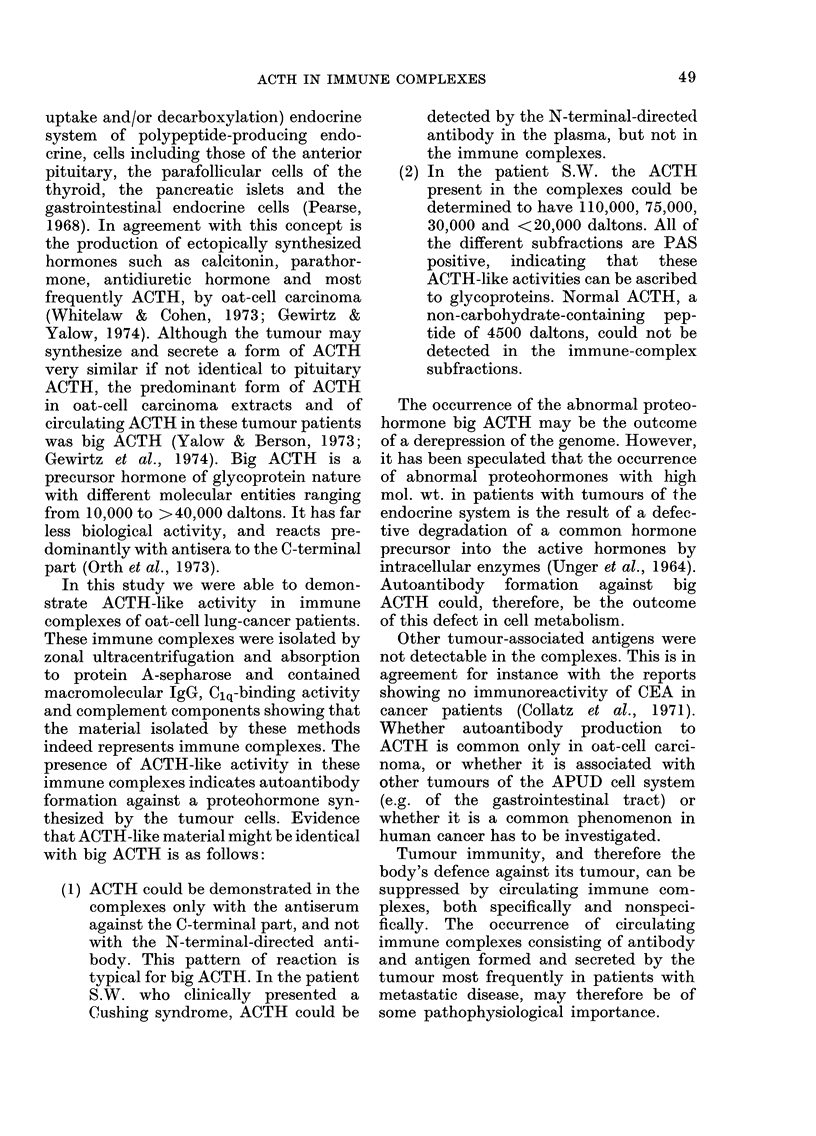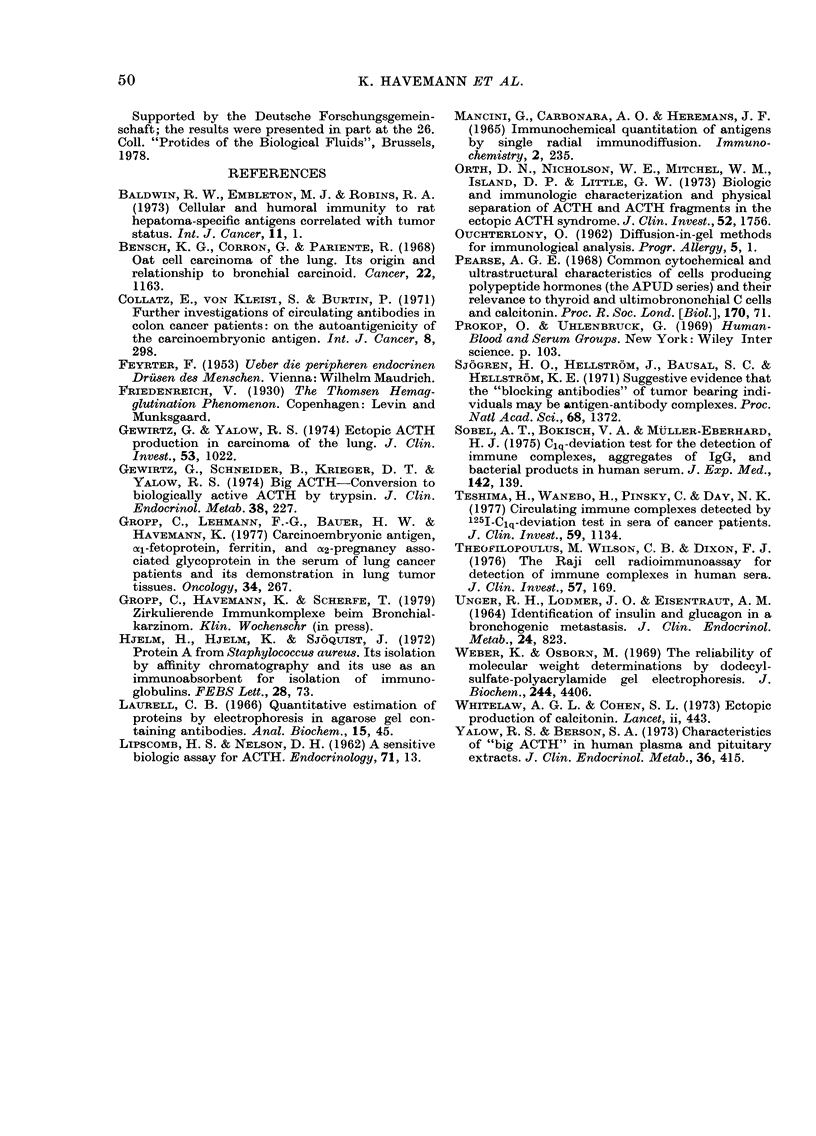# ACTH-like activity in immune complexes of patients with oat-cell carcinoma of the lung.

**DOI:** 10.1038/bjc.1979.6

**Published:** 1979-01

**Authors:** K. Havemann, C. Gropp, A. Scheuer, T. Scherfe, M. Gramse

## Abstract

**Images:**


*Br. J. Cancer* (1979) **39**, 43

### ACTH-LIKE ACTIVITY IN IMMUNE COMPLEXES OF PATIENTS WITH OAT-CELL CARCINOMA OF THE LUNG

K. HAVEMANN, C. GROPP, A. SCHEUER, T. SCHERFE AND M. GRAMSE

*From the Department of Medicine, University of Marburg, Marburg, W. Germany*

Received 7 August 1978 Accepted 17 September 1978

**Summary.—Immune complexes could be isolated from sera of 7 patients with oat-cell carcinoma of the lung, but not from 5 normal controls, using zonal ultracentrifugation. After ultracentrifugation, fractions containing macromolecular IgG were absorbed on a protein A-sepharose column and the immune complexes were eluted and dissociated by glycin-HCl buffer at pH 3·5. The eluates were tested for the presence of tumour-associated proteins as carcinoembryonic antigen (CEA), non-specific crossreacting antigen (NCA), $\alpha_2$ pregnancy associated antigen ($\alpha_2$PAG) and isoferritin. Whereas none of these tumour-associated antigens could be demonstrated, an ACTH-like activity was detected in the immune-complex fractions of 4 patients with oat-cell carcinoma, by radioimmuno- and bioassay. Polyacrylamide electrophoresis of an immune-complex fraction from a patient with Cushing syndrome showed ACTH-like activities, with mol. wt of 110,000, 75,000, 30,000 and $<$20,000 (all glycoproteins) indicating the presence of different subfractions of big ACTH.**

SOLUBLE immune complexes detected by various methods have been described in high frequency in the circulation of patients with malignant disease (Theofilopoulos *et al.*, 1976; Teshima *et al.*, 1977; Gropp *et al.*, 1979). They are associated mainly with disseminated malignancy, especially in patients with bronchial carcinoma (Teshima *et al.*, 1977; Gropp *et al.*, 1979). These findings are parallel to results obtained in animal tumour systems, showing that tumour-specific antigen-antibody complexes occur in the circulation at the time of tumour spread (Sjögren *et al.*, 1971; Baldwin *et al.*, 1973). Interference with cell-mediated immunity by humoral factors, such as tumour antigen, antibody or immune complexes has been well established. These studies demonstrate that tumour immunity can be suppressed by immune complexes in either a specific or nonspecific manner (Sjögren *et al.*, 1971, Baldwin *et al.*, 1973). In human cancer the identity of the antigens present in circulating immune complexes is unknown. One might speculate that the complexes contain tumour-specific antigens or tumour-associated carcinoembryonic antigens. Whereas up to now there is no evidence for tumour-specific antigens in man, a number of tumour-associated proteins, such as carcinoembryonic antigen (CEA), $\alpha_1$-fetoprotein (AFP), ferritin, alkaline phosphatase and several proteohormones have been associated with human malignancy (Gropp *et al.*, 1977).

It was the purpose of this study to isolate circulating immune complexes from sera of patients with disseminated oat-cell carcinoma of the lung and to identify the immune complex antigen(s). This tumour was investigated since large quantities of circulating complexes and some of the above-mentioned tumour-associated antigens can be detected in the serum of the patients.

### MATERIALS AND METHODS

$C_{1q}$-binding activity was determined with sensitized sheep erythrocytes according to Sobel *et al.* (1975). The complement components $C_3$ and $C_4$ were detected immunologically by the Mancini technique (Mancini *et al.*, 1965) or by Ouchterlony's method (1962) using monospecific antisera from Behringwerke Company. Carcinoembryonic antigen (CEA) was measured by the CIS radioimmunoassay (Isotopendienst West, Sprendlingen, W. Germany) and non-specific crossreacting antigen (NCA) was determined

FIG. 1.—Elution profile ($E_{280}$——, IgG – – –) of sera from one normal control (RE) and 7 patients with oat-cell carcinoma after zonal ultracentrifugation on 5–40% sucrose. The position of the marker proteins albumin, IgG, $\alpha_2$-macroglobulin and IgM are given with the RE control. Pool fractions |A.| |B| and |C| including their IgG content are indicated.

by radioimmunoassay*, whereas isoferritin (antiserum against isoferritin from human placenta, Behringwerke, Marburg) and $\alpha_2$-pregnancy-associated antigen ($\alpha_2$PAG) were tested by Laurell electrophoresis (Laurell, 1966). T antigen of Thomsen and Friedenreich (Friedenreich 1930; Prokop & Uhlenbruck, 1969) was determined by inhibition of anti-T *Arachis hypogaea* and anti-A *Helix pomatia* tested on neuraminidase-treated erythrocytes†. Screening for alkaline phosphatase activity was performed by the standard technique (Boehringer Company, Heidelberg) and ACTH was tested both immunologically (radioimmunoassay Amersham–Buchler with antiserum against the N-terminal part; radioimmunoassay Isotopendienst West with antiserum against the C-terminal part) and biologically by measuring the serum corticosterone release in rats (weight 180 g) after injection of 0·2 ml of immune-complex fractions. Rats receiving an injection of 0·2 ml of 0·9% NaCl served as controls (Lipscomb & Nelson 1962).

*Ultracentrifugation.*—80–150 ml of serum of 5 healthy blood donors and 7 patients with metastatic oat-cell carcinoma of the lung was subjected to zonal ultracentrifugation on a linear sucrose gradient (5–40% sucrose in 0·1M Tris HCl-buffer, pH 7·4) employing the Ti 15 rotor of a Spinco Beckman centrifuge (27,000 rev/min, 20 h, 4°C). After centrifugation, 10 ml fractions were collected and recorded for optical density (at 280 nm). Single fractions were tested for IgG (IgG Partigen plates, Behringwerke Marburg) by the Mancini technique (Fig. 1). The fractions containing monomeric IgG and macromolecular IgG (>300,000 daltons) were pooled (pool size 200–300 ml) and absorbed to protein A-sepharose (column 30 × 5 cm, Pharmacia, Uppsala, Sweden), which specifically binds IgG *via* Fc receptor (Hjelm *et al.*, 1972). After extensive washing of the absorbed material, elution and dissociation of the bound complexes were performed by 0·1M Glycin HCl-buffer, pH 3·5. The dissociated complexes eluted from protein A were dialysed against phosphate-buffered saline, pH 7·4, concentrated to 40 ml and tested for IgG, binding of $C_{1q}$, complement components and tumour-associated antigens. In addition, in one patient (S.W.) the eluted material was separated again by zonal ultracentrifugation. Based on optical density, pool fractions were prepared and investigated for the different proteins described above.

*Polyacrylamide-gel electrophoresis.* — This was performed in the presence of sodium dodecyl sulphate (SDS) according to Weber & Osborn (1969) using 7·5% gels for non-reduced and reduced and alkylated (0·1M dithioerythrol, 0·01M iodoacetamide, Serva GmbH) samples. The gels were subjected to electrophoresis at 8 mA/gel for 5 h and stained after fixation for 1 h at 60°C with 12·5% trichloroacetic acid (TCA) with Coomassie Brilliant Blue G 250 in 7·5% acetic acid or for PAS-positive material using the periodic acid–Schiff's reagent. Three gels, run parallel, were cut in lengths of 6 mm and eluted by Veronal buffer, pH 8·4, for 24 h. The eluates were filtered and then tested for ACTH by radioimmunoassay.

### RESULTS

### *Tumour-associated antigens and proteins in patients' sera*

In patients' sera but not in normal controls elevated levels of $C_{1q}$-binding activity, of CEA, ferritin and $\alpha_2$ PAG were found (Table 1). In addition, some patients showed elevated ACTH levels using the radioimmunoassay with antisera against the C-terminal part; the test performed with the N-terminal-directed antiserum was positive only in patient S.W.

### *Characterisation of isolated immune complexes*

The pool fractions containing monomeric IgG and macromolecular IgG (>300 000 daltons) were absorbed to protein A-sepharose, eluted at acid pH and concentrated to 40 ml (Fraction A, B and C). The monomeric IgG fractions (Fractions A) were negative for $C_4$ and $C_3$ and exhibited no $C_{1q}$-binding activity. The immune complex Fractions B and C after protein A absorption (Table II) contained polyclonal IgG (reactive with anti-$\kappa$ and anti-$\lambda$ light-chain antisera), $C_{1q}$-binding activity and $C_4$ in each patient.

* Kindly performed by Dr v. Kleist, Villejuif, France.
† Kindly performed by Professor K. Fischer, University Children's Hospital, Hamburg.

TABLE I.—*$C_{1q}$-deviation and tumour-associated antigens in sera of patients with oat-cell carcinoma*

| | $C_{1q}$-dev. (%) | Ferritin (μg/ml) | $\alpha_2$ PAG (mg%) | CEA (ng/ml) | ACTH (pg/ml) |
|---|---|---|---|---|---|
| Normal levels | up to 10 | 0 | 0·1 | 10 | 50 |
| Patients | | | | | |
| He | 18 | 5 | 3·5 | 19 | 70 |
| S.W. | 28 | 8 | 1·2 | 32 | >800 |
| Gb | 12·5 | 12 | 0·8 | 45 | 40 |
| Ro | 17 | 3 | 2·6 | 150 | 90 |
| Rh | 29 | 4 | 1·9 | 21 | 6 |
| Sch | 26 | 4 | 1·3 | 30 | 12 |
| Fr | 13 | 1 | 2·2 | 57 | 100 |

TABLE II.—*Demonstration of $C_{1q}$-deviation, protein, IgG, $C_4$ and ACTH (radioimmunoassay and bioassay) concentration in serum fractions of patients with lung cancer after zonal ultracentrifugation and protein A-sepharose absorption chromatography*

| Patient | Fraction | $C_{1q}$-deviation (%) | Protein (mg/ml) | IgG (mg/ml) | $C_4$ | ACTH (pg/ml) (radioimmuno-assay)* | ACTH (biol.)† |
|---|---|---|---|---|---|---|---|
| He | A | 0 | 1·5 | 1·1 | 0 | 0 | 10 |
| | B | 12·3 | 0·95 | 0·6 | 0 | 350 | 40 |
| | C | 34·1 | 0·16 | 0·1 | + | 490 | 56 |
| S.W. | A | 0 | 6·0 | >2·0 | 0 | 20 | 36 |
| | B | 21·6 | 1·0 | 0·24 | + | 255 | 100 |
| | C | 47 | 2·0 | 1·5 | + | 490 | 95 |
| Gb | A | 0 | 0·9 | 0·6 | 0 | 0 | 0 |
| | B | 12·3 | 0·45 | 0·26 | 0 | 15 | 0 |
| | C | 15 | 0·08 | 0·12 | 0 | 130 | 50 |
| Ro | A | 0 | 0·85 | 0·55 | 0 | 0 | 0 |
| | B | 18 | 0·78 | 0·4 | + | 10 | 20 |
| | C | 18·6 | 0·19 | 0·15 | + | 90 | 55 |
| Rh | A | 0 | 0·7 | 0·6 | 0 | 0 | 0 |
| | B | 12 | 0·45 | 0·4 | + | 0 | 0 |
| | C | 23·5 | 0·12 | 0·1 | 0 | 0 | 0 |
| Sch | A | 0 | 2·0 | 1·6 | 0 | 0 | 0 |
| | B | 19 | 0·7 | 0·5 | 0 | 40 | 0 |
| | C | 22 | 0·14 | 0·1 | 0 | 0 | 0 |
| Fr | A | 0 | 1·2 | 0·9 | 0 | 0 | 0 |
| | B | 25 | 0·6 | 0·3 | 0 | 0 | 0 |
| | C | 18·4 | 0·1 | 0·08 | + | 0 | 0 |

* Levels >50 pg/ml were considered to be positive.

† % release of corticosterone in relation to controls. A release of 50% or more was considered to be significant.

$C_3$ was only detectable in trace amounts in some patients. Beside these proteins no other serum proteins could be detected by immunoelectrophoresis with anti-human serum.

Strikingly, ACTH-like activity (using the radioimmunoassay with antiserum against the C-terminal part) could be detected in the complex fractions of 4/7 patients with oat-cell carcinoma. In addition, biological ACTH activity was present in the complex fractions of these 4 patients. All monomeric IgG fractions (Fractions A) from normal controls, as well as from the different patients, were negative for ACTH. Testing the immune-complex fractions after protein A with the different sensitive assays for the other tumour-

| | A | B | C I | C II | C III | C IV | C V |
|---|---|---|---|---|---|---|---|
| Vol (ml) | 10 | 10 | 4 | 4 | 4 | 4 | 4 |
| Protein (mg/ml) | 2.4 | 0·4 | 7·0 | 12·0 | 0·5 | 0·1 | 0·15 |
| CEA | 0 | 0 | 0 | 0 | 0 | 0 | 0 |
| NCA | 0 | 0 | 0 | 0 | 0 | 0 | 0 |
| $\alpha_2$ PAG | 0 | 0 | 0 | 0 | 0 | 0 | 0 |
| Alk. Phosph. (u/l) | 0 | 0 | 10 | 8 | 7 | 7 | 0 |
| Ferritin | 0 | 0 | 0 | 0 | 0 | 0 | 0 |
| T-Antigen | 0 | 0 | 0 | 0 | 0 | 0 | 0 |
| ACTH (RIA, pg/ml) | 0 | 255 | 320 | 380 | 360 | 490 | 420 |
| ACTH (Biol.) (Corticosterone % increase to controls) | 36 | 100 | 43 | 164 | 88 | 40 | 90 |
| $C_4$ | 0 | + | + | 0 | 0 | + | + |

FIG. 2.—Elution profile ($E_{280}$) of the IgG fractions A, B and C (see Fig. 1) after protein A absorption and zonal ultracentrifugation of patient SW (oat-cell carcinoma, Cushing syndrome). Volume, content of protein, alkaline phosphatase and ACTH and the presence of different tumour-associated antigens, $C_4$ and ferritin in the concentrated pool fractions are indicated below.

FIG. 3.—Separation of immune-complex Fraction C protein A II from patient SW by SDS polyacrylamide-gel electrophoresis, under non-reducing (right) and reducing (left) conditions. ACTH content, positive PAS staining (←—) and mol. wts. are indicated.

associated antigens, neither CEA, NCA, ferritin, $\alpha_2$ PAG, alkaline phosphatase nor T antigen could be detected, even in trace amounts.

A patient with metastatic oat-cell carcinoma (S.W.) who was the only one with a Cushing syndrome, revealed an unusually high amount of immune complexes (in Fraction B as well as in Fraction C). It was therefore possible to perform an additional zonal ultracentrifugation of the desorbed complexes from protein A (Fig. 2). After additional ultracentrifugation, one peak was obtained from Fractions A and B, whereas Fraction C could be separated into 5 different subfractions, all reacting positively with anti-IgG, in the $C_{1q}$-deviation test and in the ACTH radioimmuno- and bioassay. $C_4$, however, could only be demonstrated in the subfractions I, IV and V. The B fraction but not the A fraction was positive for ACTH (immunologically and biologically), for IgG, for $C_{1q}$-binding activity and $C_4$.

The subfraction II of Fraction C, containing the bulk of bioactive ACTH-like activity, was subjected to polyacrylamide-gel electrophoresis (Fig. 3). After separation the gel was cut and the different fractions were eluted and tested for ACTH-like activity. In the non-reduced samples ACTH-like activities could be demonstrated in regions showing a mol. wt. of $>60,000$ (PAS positive), 30,000 (PAS positive) and $<20,000$ (PAS positive). In the reduced samples ACTH-like activity was again present in regions of $>60,000$ and $<20,000$ daltons. The activity with 30,000 daltons disappeared. After reduction 2 protein bands with ACTH-like activity remained in the high-mol.-wt. regions (75,000 and 110,000). Simultaneously 2 strongly stained protein bands without ACTH-like activity appeared in regions with mol. wt. of about, 30,000 and 50,000. This is probably due to the reduction of IgG into heavy and light chains.

### DISCUSSION

About one-third of all malignant pulmonary tumours are oat-cell carcinomas. Thus, at least a third of the ~100,000 deaths annually attributable to lung carcinoma in the United States are caused by this neoplasm, which inevitably leads to death within a short period. The presence of characteristic neurosecretory-type granules within oat-cell tumours of the lung suggests that so called K-cells (Kulchinsky cells) of the bronchial mucosa are the physiological counterpart of this tumour (Bensch *et al*, 1968). Feyrter was the first to associate K-cells with an endocrine function which he considered to be part of a diffuse "endocrinic epithelial organ" (Feyrter, 1953). An extension of this postulate combined the K-cells with the so-called APUD (amine precursor uptake and/or decarboxylation) endocrine system of polypeptide-producing endocrine, cells including those of the anterior pituitary, the parafollicular cells of the thyroid, the pancreatic islets and the gastrointestinal endocrine cells (Pearse, 1968). In agreement with this concept is the production of ectopically synthesized hormones such as calcitonin, parathormone, antidiuretic hormone and most frequently ACTH, by oat-cell carcinoma (Whitelaw & Cohen, 1973; Gewirtz & Yalow, 1974). Although the tumour may synthesize and secrete a form of ACTH very similar if not identical to pituitary ACTH, the predominant form of ACTH in oat-cell carcinoma extracts and of circulating ACTH in these tumour patients was big ACTH (Yalow & Berson, 1973; Gewirtz *et al.*, 1974). Big ACTH is a precursor hormone of glycoprotein nature with different molecular entities ranging from 10,000 to >40,000 daltons. It has far less biological activity, and reacts predominantly with antisera to the C-terminal part (Orth *et al.*, 1973).

In this study we were able to demonstrate ACTH-like activity in immune complexes of oat-cell lung-cancer patients. These immune complexes were isolated by zonal ultracentrifugation and absorption to protein A-sepharose and contained macromolecular IgG, $C_{1q}$-binding activity and complement components showing that the material isolated by these methods indeed represents immune complexes. The presence of ACTH-like activity in these immune complexes indicates autoantibody formation against a proteohormone synthesized by the tumour cells. Evidence that ACTH-like material might be identical with big ACTH is as follows:

(1) ACTH could be demonstrated in the complexes only with the antiserum against the C-terminal part, and not with the N-terminal-directed antibody. This pattern of reaction is typical for big ACTH. In the patient S.W. who clinically presented a Cushing syndrome, ACTH could be detected by the N-terminal-directed antibody in the plasma, but not in the immune complexes.

(2) In the patient S.W. the ACTH present in the complexes could be determined to have 110,000, 75,000, 30,000 and <20,000 daltons. All of the different subfractions are PAS positive, indicating that these ACTH-like activities can be ascribed to glycoproteins. Normal ACTH, a non-carbohydrate-containing peptide of 4500 daltons, could not be detected in the immune-complex subfractions.

The occurrence of the abnormal proteohormone big ACTH may be the outcome of a derepression of the genome. However, it has been speculated that the occurrence of abnormal proteohormones with high mol. wt. in patients with tumours of the endocrine system is the result of a defective degradation of a common hormone precursor into the active hormones by intracellular enzymes (Unger *et al.*, 1964). Autoantibody formation against big ACTH could, therefore, be the outcome of this defect in cell metabolism.

Other tumour-associated antigens were not detectable in the complexes. This is in agreement for instance with the reports showing no immunoreactivity of CEA in cancer patients (Collatz *et al.*, 1971). Whether autoantibody production to ACTH is common only in oat-cell carcinoma, or whether it is associated with other tumours of the APUD cell system (e.g. of the gastrointestinal tract) or whether it is a common phenomenon in human cancer has to be investigated.

Tumour immunity, and therefore the body's defence against its tumour, can be suppressed by circulating immune complexes, both specifically and nonspecifically. The occurrence of circulating immune complexes consisting of antibody and antigen formed and secreted by the tumour most frequently in patients with metastatic disease, may therefore be of some pathophysiological importance.

Supported by the Deutsche Forschungsgemeinschaft; the results were presented in part at the 26. Coll. "Protides of the Biological Fluids", Brussels, 1978.

### REFERENCES

BALDWIN, R. W., EMBLETON, M. J. & ROBINS, R. A. (1973) Cellular and humoral immunity to rat hepatoma-specific antigens correlated with tumor status. *Int. J. Cancer*, **11**, 1.

BENSCH, K. G., CORRON, G. & PARIENTE, R. (1968) Oat cell carcinoma of the lung. Its origin and relationship to bronchial carcinoid. *Cancer*, **22**, 1163.

COLLATZ, E., VON KLEIST, S. & BURTIN, P. (1971) Further investigations of circulating antibodies in colon cancer patients: on the autoantigenicity of the carcinoembryonic antigen. *Int. J. Cancer*, **8**, 298.

FEYRTER, F. (1953) *Ueber die peripheren endocrinen Drüsen des Menschen.* Vienna: Wilhelm Maudrich.

FRIEDENREICH, V. (1930) *The Thomsen Hemagglutination Phenomenon.* Copenhagen: Levin and Munksgaard.

GEWIRTZ, G. & YALOW, R. S. (1974) Ectopic ACTH production in carcinoma of the lung. *J. Clin. Invest.*, **53**, 1022.

GEWIRTZ, G., SCHNEIDER, B., KRIEGER, D. T. & YALOW, R. S. (1974) Big ACTH—Conversion to biologically active ACTH by trypsin. *J. Clin. Endocrinol. Metab.* **38**, 227.

GROPP, C., LEHMANN, F.-G., BAUER, H. W. & HAVEMANN, K. (1977) Carcinoembryonic antigen, $\alpha_1$-fetoprotein, ferritin, and $\alpha_2$-pregnancy associated glycoprotein in the serum of lung cancer patients and its demonstration in lung tumor tissues. *Oncology*, **34**, 267.

GROPP, C., HAVEMANN, K. & SCHERFE, T. (1979) Zirkulierende Immunkomplexe beim Bronchialkarzinom. *Klin. Wochenschr* (in press).

HJELM, H., HJELM, K. & SJÖQUIST, J. (1972) Protein A from *Staphylococcus aureus*. Its isolation by affinity chromatography and its use as an immunoabsorbent for isolation of immunoglobulins. *FEBS Lett.*, **28**, 73.

LAURELL, C. B. (1966) Quantitative estimation of proteins by electrophoresis in agarose gel containing antibodies. *Anal. Biochem.*, **15**, 45.

LIPSCOMB, H. S. & NELSON, D. H. (1962) A sensitive biologic assay for ACTH. *Endocrinology*, **71**, 13.

MANCINI, G., CARBONARA, A. O. & HEREMANS, J. F. (1965) Immunochemical quantitation of antigens by single radial immunodiffusion. *Immunochemistry*, **2**, 235.

ORTH, D. N., NICHOLSON, W. E., MITCHEL, W. M., ISLAND, D. P. & LITTLE, G. W. (1973) Biologic and immunologic characterization and physical separation of ACTH and ACTH fragments in the ectopic ACTH syndrome. *J. Clin. Invest.*, **52**, 1756.

OUCHTERLONY, O. (1962) Diffusion-in-gel methods for immunological analysis. *Progr. Allergy*, **5**, 1.

PEARSE, A. G. E. (1968) Common cytochemical and ultrastructural characteristics of cells producing polypeptide hormones (the APUD series) and their relevance to thyroid and ultimobrononchial C cells and calcitonin. *Proc. R. Soc. Lond.* [*Biol.*], **170**, 71.

PROKOP, O. & UHLENBRUCK, G. (1969) *Human-Blood and Serum Groups.* New York: Wiley Inter science. p. 103.

SJÖGREN, H. O., HELLSTRÖM, J., BAUSAL, S. C. & HELLSTRÖM, K. E. (1971) Suggestive evidence that the "blocking antibodies" of tumor bearing individuals may be antigen-antibody complexes. *Proc. Natl Acad. Sci.*, **68**, 1372.

SOBEL, A. T., BOKISCH, V. A. & MÜLLER-EBERHARD, H. J. (1975) $C_{1q}$-deviation test for the detection of immune complexes, aggregates of IgG, and bacterial products in human serum. *J. Exp. Med.*, **142**, 139.

TESHIMA, H., WANEBO, H., PINSKY, C. & DAY, N. K. (1977) Circulating immune complexes detected by $^{125}I$-$C_{1q}$-deviation test in sera of cancer patients. *J. Clin. Invest.*, **59**, 1134.

THEOFILOPOULUS, M. WILSON, C. B. & DIXON, F. J. (1976) The Raji cell radioimmunoassay for detection of immune complexes in human sera. *J. Clin. Invest.*, **57**, 169.

UNGER, R. H., LODMER, J. O. & EISENTRAUT, A. M. (1964) Identification of insulin and glucagon in a bronchogenic metastasis. *J. Clin. Endocrinol. Metab.*, **24**, 823.

WEBER, K. & OSBORN, M. (1969) The reliability of molecular weight determinations by dodecylsulfate-polyacrylamide gel electrophoresis. *J. Biochem.*, **244**, 4406.

WHITELAW, A. G. L. & COHEN, S. L. (1973) Ectopic production of calcitonin. *Lancet*, ii, 443.

YALOW, R. S. & BERSON, S. A. (1973) Characteristics of "big ACTH" in human plasma and pituitary extracts. *J. Clin. Endocrinol. Metab.*, **36**, 415.